# The Impact of Hyaluronic Acid Coating on the Cationic Niosomal Surface for Doxorubicin Delivery

**DOI:** 10.3390/molecules30051148

**Published:** 2025-03-03

**Authors:** Elisabetta Mazzotta, Martina Romeo, Giuseppina Sacco, Selene De Benedittis, Antonio Qualtieri, Ida Daniela Perrotta, Rita Muzzalupo

**Affiliations:** 1 Department of Pharmacy, Health and Nutritional Sciences, University of Calabria, 87036 Rende, Italy; mazzotta-elisabetta@libero.it (E.M.); martina.romeo@unical.it (M.R.); giusysacco27@gmail.com (G.S.); 2 Institute for the Research and the Biomedical Innovation (IRIB)-CNR-Mangone (CS), 00185 Rome, Italy; selene.db90@gmail.com (S.D.B.); antonio.qualtieri@cnr.it (A.Q.); 3 Centre for Microscopy and Microanalysis (CM2), Department of Biology Ecology and Earth Sciences, University of Calabria, 87036 Rende, Italy; ida.perrotta@unical.it

**Keywords:** niosomes, cationic lipids, CD44 receptor, targeted drug delivery systems, anticancer therapy

## Abstract

This study was designed to develop cationic vesicles for doxorubicin (DOX) delivery and to compare anticancer efficacy of these systems uncoated and coated with hyaluronic acid. Cationic nanoformulation was first optimized using various amounts of Span80, DODAB, and cholesterol. The optimized niosomal formulation (CTN4) in terms of vesicle size, surface zeta potential, and colloidal stability was coated with hyaluronic acid and the in vitro therapeutic effectiveness in uterine cervix cancer cells of vesicles loaded with DOX was tested. In vitro studies revealed significantly superior cytotoxicity against Hela cells of niosomes coated with HA compared to uncoated formulations. Moreover, cytotoxicity was also evaluated on normal fibroblast murine cell line, NIH-3T3 cells, and the results obtained demonstrated that HA-coated vesicles exhibited lower cytotoxicity to NIH-3T3 cells compared to uncoated nanovesicles. These findings highlighted how the surface coating influences the effectiveness of niosomes developed as a target drug delivery system and the selectivity and the antitumour efficacy of chemotherapeutic drugs.

## 1. Introduction

The development of a highly effective therapeutic systems with low side effects requires the specific drug delivery target site. For this purpose, smart nanocarriers have been intensively studied to specifically deliver therapeutic agents to a particular site within the body, such as a tumoral or inflamed tissue. Various approaches such as passive targeting, active targeting, and multi-responsivity have been developed to provide cancer cell specificity to nanocarriers, aiming to enhance the efficacy of drugs while minimizing side effects and reducing systemic toxicity [1,2,3,4]. Specifically, active targeting refers to a strategy aimed to selectively accumulate therapeutic or imaging agents at a target site by incorporating targeting ligands onto the surface of nanoparticles or other carrier systems [5,6]. These ligands can specifically bind to receptors or molecules overexpressed on the surface of target cells or tissues, allowing the specific delivery and localization of the therapeutic. Active targeting enhances the efficiency of drug delivery, reduces off-target effects, and improves the overall efficacy of the treatment. Common targets employed in cancer therapy are enzyme and receptors overexpressed in tumour cells such as transferrin receptor (TfR) [7], human epidermal growth factor receptor-2 [8], folic acid receptor (FaR) [9], and CD44 receptor [10].

Hyaluronic acid (HA), a main component of the extracellular matrix, is a biocompatible and biodegradable hydrophilic polymer able to bind to CD44 receptor specifically overexpressed in many solid tumours, including lung, breast, gastric, cervical, kidney, and liver cancer cells [11]. Moreover, CD44 appears to play a role in regulating the proliferation of cancer cells and lymphocyte adhesion during migration, a process similar to metastatic dissemination of solid tumours [12]. Indeed, HA has been extensively employed as a drug carrier or targeting component for nanoparticles aimed at targeting cells overexpressing CD44 [13,14]. Active targeting strategies are successfully used to gain selectivity to cationic nanocarriers, which, for their ability to interact with cellular membranes possessing anionic properties, are delivered to different organs or cells without specific targeting. In recent years, there has been considerable interest in cationic vesicles, especially liposomes. However, fewer studies have focused on cationic niosomes, even though they offer several advantages, including lower production costs, greater stability, and reduced toxicity [15,16,17]. Moreover, niosomes are composed of chemically stable nonionic surfactants that are less susceptible to hydrolysis or oxidation and are able to improve the stability at different environmental conditions when compared to the phospholipid bilayer of liposomes. These nanodevices are usually formed through the self-assembly of non-ionic surfactant molecules and one or more cationic lipids. Recently, cationic niosomes have gained a large application in gene therapy for their ability to enhance the stability and delivery of negatively charged nucleic acids [18]. Moreover, cationic vesicles have been widely employed in selective drug delivery to solid tumors thanks to their ability to preferentially target the angiogenic endothelium of solid tumor via electrostatic interactions with anionic molecules overexpressed in tumor vasculature than other tissues [19,20,21]. Anyway, cationic vesicles are recognized for inducing toxicity in macrophages, macrophage-like cells, and monocyte-like cells and for modifying the secretion of crucial immuno-modulators [22,23,24]. Furthermore, following intravenous administration, cationic nanoparticles are sequestered in the organs of the reticuloendothelial system, such as the spleen and liver leading to significant toxicity in these organs and impairing proper immune system function.

For instance, Kedmi et al. [25] reported that injecting cationic liposomes containing dioleoyl-3-trimethylammonium propane (DOTAP) induced higher in vivo hepatotoxicity and proinflammatory responses compared to neutral and negatively charged liposomes. Moreover, an important cytotoxicity of the synthetic cationic lipid dioctadecyldimethylammonium bromide (DODAB) widely employed as both a carrier and an adjuvant in immunological applications has been reported [26,27,28]. Consequently, addressing the cytotoxicity of cationic lipids is crucial and various strategies have been developed for this purpose, such as combining them with other lipids [29] or coating with polymers [30].

Herein, we developed cationic niosomes made up of Span 80 (S80), cholesterol (chol), and DODAB. The best formulation was characterized and evaluated in both uncoated and HA-coated forms to investigate the tumor-targeting potential of these systems for doxorubicin (DOX) delivery. In fact, intravenous administration of free DOX results in nonspecific toxic effects in various tissues, not just in the target tumor. Therefore, designing carriers that can prevent or minimize the distribution of DOX to non-target tissues and promote selective, controlled drug release within the tumor is crucial for its therapeutic effectiveness. To trial our niosomal carriers, in vitro cytotoxicity and uptake studies were performed on normal and tumor cells to verify the efficacy of the two different targeting strategies for DOX release.

## 2. Results and Discussions

Cationic vesicles are a promising carrier system for delivering anticancer agents to tumor endothelial cells due to their intrinsic targeting ability, which stems from the natural affinity of cationic molecules on the vesicle surface for anionic molecules in the tumor microvasculature [31,32]. Moreover, HA-based nanodevices have been widely studied as tumor target delivery systems for their ability to increase drug concentration in tumor cells that overexpressed CD44 receptors.

In the present study, we developed cationic niosomes for DOX delivery and tested the anticancer efficacy of the uncoated and HA-coated system. Subsequently, we compared the efficacy of these two different targeting approaches to increase the anticancer efficacy of DOX in Hela tumor cell lines and, at the same time, reduce the toxicity on normal NIH-3T3 cells.

To realize cationic vesicles, the lipid DODAB has been chosen and used in combination with S80 and Chol. DODAB characterized by a positively charged quaternary ammonium head group is known to form bilayer structures [33]. DODAB are indeed widely used in the development of gene carriers for its physical properties and the ability to interact with DNA [34,35,36].

Furthermore, cationic DODAB has shown promising results as vaccine immunoadjuvants [37,38,39]. To assess the impact of DODAB on the surface charge of niosomes, varying quantities of DODAB were used, developing four different formulations based on S80 and different amounts of Chol. Cationic niosomes developed displayed appropriate nano-scaled size in the range between 431–576 nm and a narrow size distribution with polydispersion index (PI) < 0.3.

Both size and ζ potential (ZP) play crucial roles in monitoring the shelf-life stability of vesicles. Smaller niosomes with elevated electrical surface potential tend to exhibit greater stability. Table 1 shows how the ZP change as a function of DODAB content. The ratio between DODAB moles and the sum of other lipids, Span80 and cholesterol, used in niosome system preparation is referred to as R in Table 1. The zeta potential sign can only be reversed if the R value is higher than 0.20. Only the formulations CTN3 and CTN4 containing 8 mg DODAB exhibited high positive ZP (Figure S1A).

Moreover, the formation of a precipitate, probably dependent on the high cholesterol level, was already observed overnight for CTN3, while any precipitate occurred for CTN4. Moreover, the colloidal stability of CTN4 in terms of vesicle size, PI, and ZP was tested by storing them at room temperature. After storage for 4 months, the vesicle size, PI, and ZP were not significantly changed from the freshly prepared samples (Table 2). These results confirmed the high colloidal stability of the cationic niosomes developed. Based on these results, CTN4 sample was chosen as the formulation for further studies.

HA has been coated on the niosome surface to improve their stealth properties and to provide targeting for CD44-positive cancer cells. The successful conjugation via electrostatic interaction of HA was confirmed by the slight increase in particle size and by the significant inversion of ZP values, as observed in Table 3.

**Figure 1:**
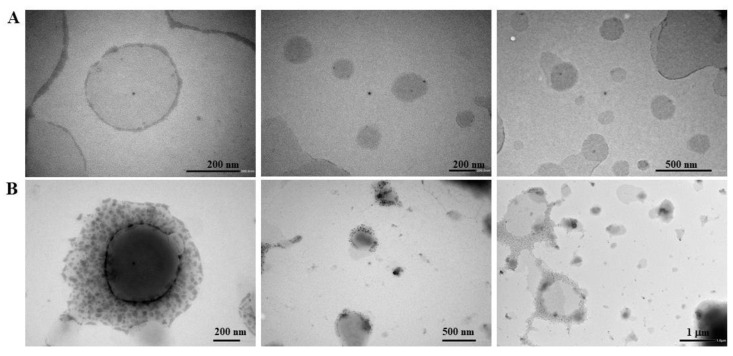
Typical TEM photomicrograph of CTN4 (**A**) and CTN4-HA (**B**) formulations.

The increase in hydrodynamic size of vesicles coated with HA is probably due to the fact that HA is a hydrophilic molecule, which adsorbs water and, as reported in the literature, can swell in aqueous media [40]. Moreover, the ZP directly converted from a positive charge of +43.9 ± 0.7 to a negative value of −37.7 ± 0.6 after incubation with HA due to the carboxylic negative residues of HA (Figure S1B). This inversion in surface charge confirmed that HA molecules are conjugated on niosomes by electrostatic interaction. The high values of ZP highlighted the high colloidal stability of the formulation developed. The effective coating was also proved by transmission electron microscope (TEM) analysis that was utilized to characterize the particle morphology of both uncoated (Figure 1A) and HA-coated (Figure 1B) CTN4 formulations. The micrograph illustrates the spherical shape and smooth surface of the nanoniosomes, indicating a narrow size distribution in the nanometer scale, which was consistent with that measured by the dynamic light scattering method. Additionally, Figure 1B demonstrates the presence of a distinct coating layer formed by HA on the surface of the vesicles, which was absent in noncoated vesicles. These fluffy dark structures suggest that molecules of HA were exposed on the vesicle surface, as also previously reported in the literature [41,42].

**Figure 2:**
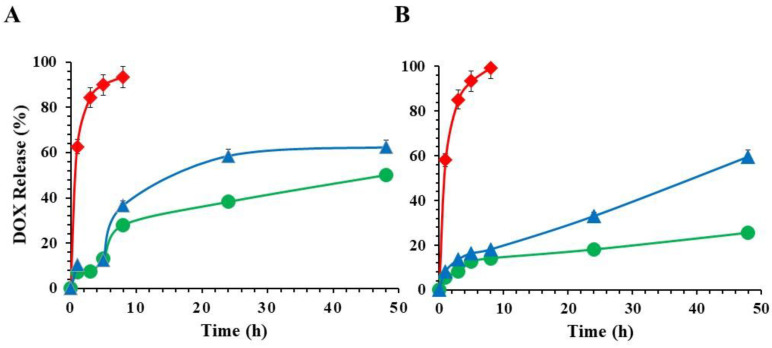
Drug release profiles of DOX from DOX-CTN4 (green), DOX-CTN4-HA (blue), and DOX solution (red) at pH 5.5 (**A**) and 7.4 (**B**) at 37 °C (mean ± standard deviation, *n* = 3).

The DOX entrapment efficiency was 80.5 ± 4.3%. The drug release properties of niosomes were investigated using the dialysis method under different pH conditions. As shown in Figure 2, the DOX solution reached full release after 8 h at both pH, whereas the release of DOX from niosomes was delayed and slower, resulting in a sustained release process without any burst release. This suggests that the vesicle bilayer acts as a barrier to the drug. Furthermore, a more controlled release of the drug is observed in cationic formulations. This may be due to the fact that cationic lipids cause a more stringent molecular arrangement in the bilayer, resulting in slower drug release. Diffusion thereby prevents the premature release of DOX, as also already reported in the literature [43,44,45]. Moreover, the permeability of cationic drugs is significantly reduced if the vesicle layer possessed a positive surface potential. The positive surface potential due to the presence of DODAB led to the formation of electrostatic repulsion with DOX, resulting in a reduction in the drug concentration gradient across the membrane and a reduction in drug partitioning into the bilayer. At pH 5.5, the DOX release is approximately 50% and 60% from the CT4N and CT4N-HA formulations, respectively, while, at pH 7.4, the drug release is lower in the CT4N formulation and remains almost unchanged in the one coated with HA.

The compatibility of carriers with biological systems is crucial for their use as carriers for cancer therapy when administered systemically before clinical application. Hence, preliminary assessment of biocompatibility of all prepared formulations was conducted by determining the hemolytic effect on human blood, using distilled water as a positive control and PBS as a negative control. It was observed that the hemolytic activity of CTN4 and CTN4-HA systems remained below 5%, which is an acceptable limit, at a concentration of niosomes up to 126 μM, indicating satisfactory biocompatibility. However, for the systems, DOX-CTN4, a hemolytic activity equal to 10.44% was observed. It is widely recognized, in fact, that cationic nanocarriers carry the risk of increased cytotoxicity and interaction with serum proteins, limiting their potential in vivo application [46,47]. Anyway, the coating with hydrophilic and anionic shielding polymers, such as HA in DOX-CTN4-HA formulation, is able to counteract this minimal toxicity by bringing the hemolytic activity to values lower than 5% (specifically 4.06%).

### 2.1. In Vitro Cytotoxicity Activity

The in vitro cytotoxicity of DOX-CTN4 formulations in HeLa and NIH-3T3 cells was evaluated by the MTT assay.

DOX loading into both coated and uncoated CTN4 vesicles leads to an increase in antitumor activity in HeLa cells with respect to the free drug. Specifically, at the low concentrations tested (0.1 and 1 µM), vesicles coated with HA (CTN4-HA) show greater toxicity compared to uncoated systems (CTN4). For instance, Hela cell viability was found to be 59.57% and 41.42% for cells treated with uncoated and coated systems at a concentration of 0.1 µM, respectively. Additionally, at this concentration, the cytotoxic effect of DOX when encapsulated in coated niosomes was significantly higher compared to the free form, which was only 82.45% (*p* < 0.05). These results demonstrated that the selectivity of CTN4-HA was better than that of free DOX, highlighting the targeting effect of hyaluronic acid, which promotes the internalization of nanosystems in tumor cells. However, at the highest concentration used (10 µM), any difference in anticancer efficacy was observed for CTN4 and CTN4-HA formulations, probably due to the achievement of plateau in cytotoxic activity related to HA receptor saturation.

**Figure 3:**
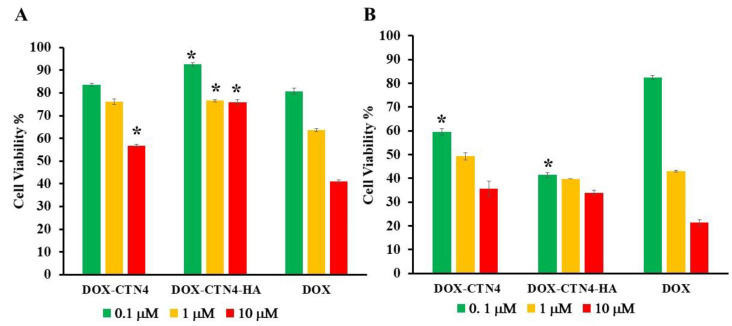
NIH-3T3 (**A**) and HeLa (**B**) cell viability tested by 3-(4,5-dimethylthiazol-2-yl)-2,5-diphenyltetrazolium bromide (MTT) assay in triplicate. Cells were incubated with DOX-CTN4 and DOX-CTN4-HA formulations for 72 h. The results are expressed as the percentage of the control assumed as 100%. Each value represents the mean ± SD of three independent experiments. * *p* < 0.05 vs. free DOX.

The toxicity of the developed systems was also evaluated on healthy cells and the results are reported in Figure 3A. DOX-CTN4-HA niosomes exhibited reduced toxicity compared to the noncoated systems. Normal cells treated with free DOX, noncoated niosomes, and coated niosomes at a concentration of 10 μM showed cell viability of 41.06%, 56.76%, and 75.84% after 72 h, respectively. These results highlight how encapsulating DOX in hyaluronic-acid-coated systems reduces toxicity on NIH-3T3 cells, which are CD44-receptor-negative, while it increases cytotoxic activity against HeLa cancer cells that overexpress this receptor.

As expected, the formulation DOX-CTN4-HA showed obviously higher cytotoxicity to HeLa cells than to NIH-3T3 cells at all the treated concentrations. The higher cytotoxicity might be attributed to the enhanced cellular uptake efficacy of DOX-CTN4-HA through CD44-receptor-mediated endocytosis into the CD44-receptor-overexpressing HeLa cells.

### 2.2. Uptake Studies

**Figure 4:**
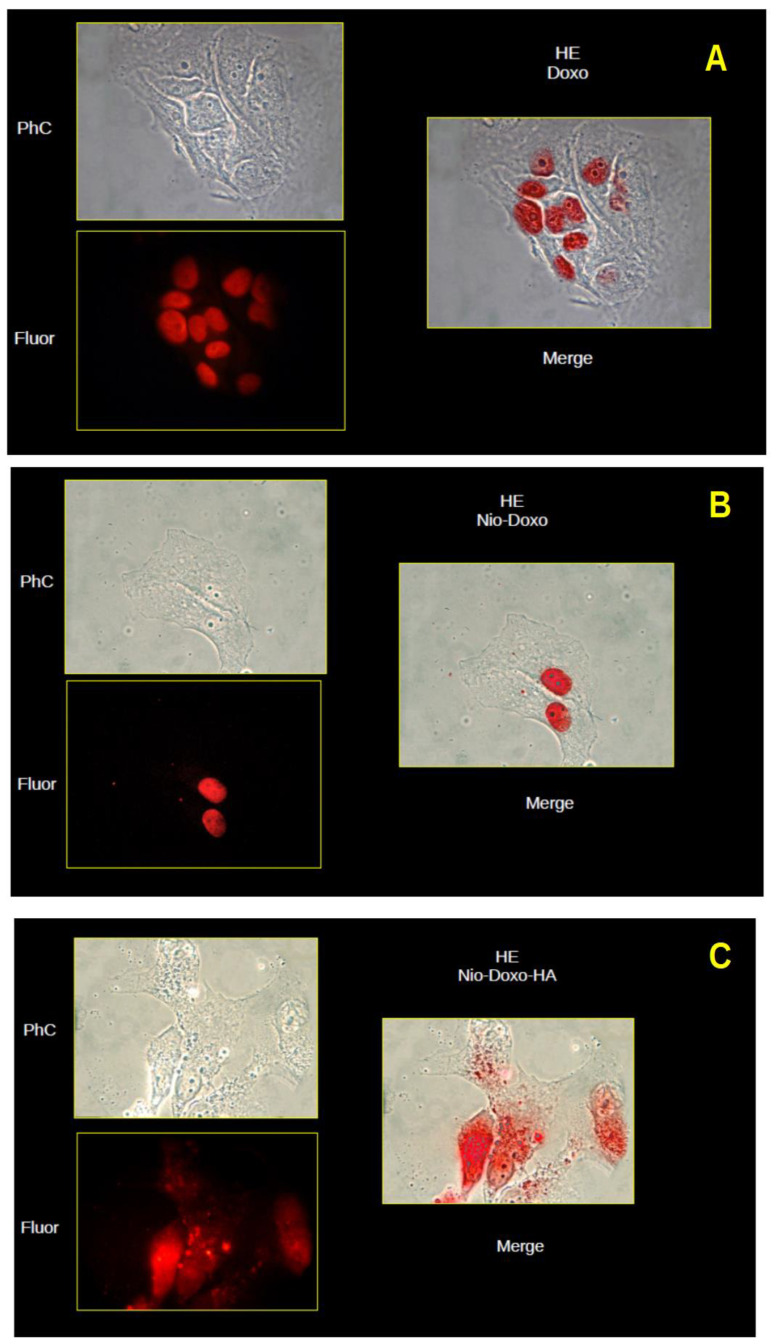
Microscopic images (40×) of HE cells in phase contrast (PhC) and fluorescence at 590 nm (Fluor). Merge of the two images to the right. (**A**) DOX solution, (**B**) DOX-CTN4 and (**C**) DOX-CTN4-HA, respectively.

**Figure 5:**
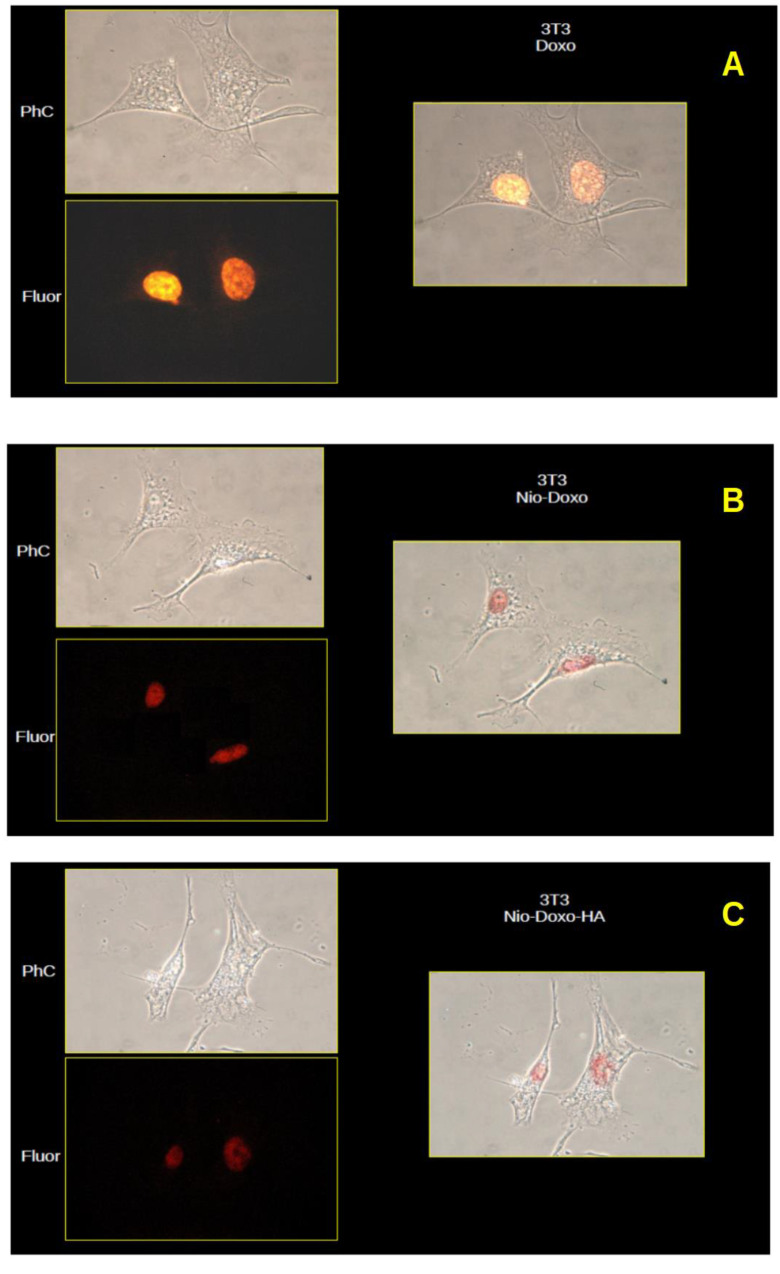
Microscopic images (40×) of NIH-3T3 cells in phase contrast (PhC) and fluorescence at 590 nm (Fluor). Merge of the two images to the right. (**A**) DOX solution, (**B**) DOX-CTN4 and (**C**) DOX-CTN4-HA, respectively.

Cellular uptake was studied by examining the intracellular localization of DOX, administered both free and with the two types of nanoparticles used as a vehicle. Due to its fluorescent nature, DOX can be used to track the internalization and intracellular localization of nanoparticles. The NIH-3T3 cell we observed showed DOX fluorescence only at the nuclear level for all three types of administration, both free DOX and the two niosome formulations, although with different intensity: HeLa cells showed only nuclear fluorescence when DOX was administered free and with CTN4, while, when cells were treated with DOX-CTN4-HA nanoparticles, the fluorescence apparently appeared more intense with the same exposure times and photographic capture parameters used but, above all, it was possible to observe fluorescent images at the cytoplasmic and perinuclear level, which were not observable in the NIH-3T3 cells (Figure 4 and Figure 5). Especially on the basis of this last observation, we could hypothesize that the uptake of CTN4-HA particles in HeLa cells could involve some different mechanism, such as the receptor one when compared to 3T3 cells.

## 3. Experimental Section

### 3.1. Materials

Span 80 (S80), cholesterol (Chol), dimethyldioctadecylammonium bromide (DODAB), Hyaluronic acid 1200–1800 kDa (HA), doxorubicin (DOX), tetrazolium salt (3-(4,5-dimethylthiazol-2-yl)-2,5-diphenyltetrazolium bromide or MTT, ethylenediaminetetraacetic acid (EDTA), phosphate buffer pH 7.4, and all solvents used were bought from Sigma-Aldrich (Milan, Italy). Dulbecco’s modified Eagle’s medium (DMEM) fetal bovine serum (FBS), and l-glutamine were purchased (Thermo-Fischer Scientific, Waltham, MA, USA).

### 3.2. Preparation of Cationic Niosomes

Preliminarily, experiments to optimize DODAB contents were performed to achieve a cationic formulation with a suitable colloidal stability. So, various cationic niosomes made up of Span 80 (S80), dimethyldioctadecylammonium bromide (DODAB), and cholesterol at different molar ratios were prepared by thin-layer evaporation technique according to the amount reported in Table 1.

The lipid DODAB, Chol, and S80 were dissolved in chloroform and the solvent was removed under vacuum in a rotary evaporator, forming the homogeneous lipid film. Then, lipid film was hydrated with 10 mL of distilled water or DOX aqueous solution (5.4 × 10^−4^ M) at 60 °C for 30 min. After being left overnight, the formed multilamellar vesicles were then shattered by ultrasound to form small niosomes with a uniform size.

HA coating was carried out, incubating 1 mL of CTN4 with 0.5 mL of HA aqueous solution (1 mg/mL) at room temperature for 1 h with gentle mixing at 300 rpm. The resulting CTN4- HA was kept at 4 °C.

### 3.3. Physicochemical Characterization

Dynamic light scattering (DLS) (Zetasizer Nano ZS, Malvern Instruments Ltd., Malvern, U.K) was used to assess the size, particle distribution, and ζ potential (ZP). The niosomes were diluted in distilled water (1:100), and all of the measurements were taken at 25 °C. TEM micrographs of HA-coated and uncoated niosomes were assessed for morphology and size using TEM JEOL 1400 (Jeol 1400 Plus electron microscope, JEOL Ltd., Milano, Italy), operating at an acceleration voltage equal to 80 kV. For TEM imaging, all samples were left to dry at room temperature before the microscopy observation. All measurements were conducted at room temperature.

Dialysis was used to determine the encapsulation efficiency of the drug (E%) by removing the unencapsulated substance. The dialysis process involves the passage of the unencapsulated solute through a semi-permeable membrane. Under magnetic agitation, a dialysis bag (Spectra/Por^®^, cut-off 12–14 kDa, SERVA Electrophoresis GmbH, Heidelberg, Germany) was placed in distilled water and niosomes were inserted into it, according to this method. The free drug was dialyzed for 60 min and the dialysis was finished when there was no drug detected in the receiving solution after four water changes. The encapsulation efficiency of the sample was calculated by taking into account the percentage of the drug trapped in the niosomes versus the original amount present in the non-dialyzed sample. The evaluation was performed by diluting 1 mL of dialysis niosome with 25 mL of methanol to break down the niosome membrane. The absorbance of both solutions was determined by measuring it at 495 nm using a JASCO V-530 UV-vis spectrometer (JASCO Corporation, Tokyo, Japan) with 1 cm quartz cells, which allowed for the determination of the concentration of DOX present in the solutions and the calculation of the E%. Each experiment has been run in triplicate and the results are expressed as SD mean.

The stability of CTN4 stored at room temperature was assessed by measuring sizes and ZP at specific time intervals for up to 4 months. Each measurement was conducted three times, and the results were recorded as the mean ± standard deviation.

### 3.4. Hemolytic Assay

To evaluate the hemolytic activity of the niosome formulations prepared in this study, we followed the method outlined by Pape et al. [48]. Initially, blood was centrifuged at 3000 rpm for 10 min. The supernatant was discarded, and the erythrocytes were suspended in phosphate-buffered saline solution (PBS 7.4) to eliminate white cells and other debris, followed by centrifugation at 3000 rpm for 10 min. This washing process was repeated three times. The erythrocyte stock suspension was then diluted in PBS to a concentration of 8 × 10^9^ cells/mL. Various volumes of the niosomal solution, ranging from 10 to 100 µL, were added to Eppendorf tubes. Subsequently, phosphate buffer and 25 µL of erythrocytes were added to each tube to reach a total volume of 1 mL. The samples were shaken for 10 min at room temperature, followed by centrifugation (5 min at 10,000 rpm). Hemolytic activity was assessed by comparing the absorbance value of the supernatant at 575 nm with that of control samples, which were fully hemolyzed using ultrapure water. All analyses were performed in triplicate.

### 3.5. In Vitro Release Studies

In vitro release studies of niosomal formulations were performed by dialysis method under sink conditions in two different media, acetate buffer (pH 5.5) and phosphate buffer (pH 7.4), to mimic an acidic tumor microenvironment and physiological conditions, respectively. A total of 1 mL of each sample was placed in a dialysis bag and placed under continuous stirring in 25 mL of medium. At set time points, 2 mL of the medium were withdrawn at predetermined time intervals and exchanged with the fresh medium and analyzed by UV–VIS spectrophotometry at 495 nm. The drug concentration was calculated according to the standard curve of DOX (Figure S2) and the results are reported as cumulative release (%) applying the following formula:

(1) $$DOX\;Release(\%)=\frac{\;(\mathrm{DOX}\;\mathrm{moles}\;\mathrm{released}\;\mathrm{from}\;\mathrm{niosomes})}{\left(\mathrm{initial}\;\mathrm{DOX}\;\mathrm{moles}\;\mathrm{encapsulated}\;\mathrm{in}\;\mathrm{the}\;\mathrm{niosomes}\right)}\times 100$$

Each experiment was carried out in triplicate.

### 3.6. Cell Culture

HeLa cells and NIH-3T3 cells obtained from the American Type Culture Collection (ATCC, Manassas, VA, USA) were cultured in Dulbecco’s modified Eagle’s medium (DMEM) (Thermo-Fischer Scientific, Waltham, MA, USA), supplemented with 10% (*v*/*v*) fetal bovine serum (FBS), 2 mM l-glutamine, 100 IU/mL penicillin, and 100 µg/mL streptomycin. Cultures were maintained at 37 °C in a humidified atmosphere containing 5% CO_2_. Upon reaching approximately 80% confluence, cells were detached from 25 cm^2^ flasks using trypsin ethylenediaminetetraacetic acid (EDTA) and subcultured into 96-multiwell plates for experimental use.

### 3.7. In Vitro Cytotoxicity Assay

The in vitro cytotoxicity of the developed formulations was assessed in CD44-positive cancer cell lines (HeLa) and one CD44-negative fibroblast cell line (NIH-3T3) using the MTT assay. Initially, cells were plated at a density of 6 × 10^4^ cells/mL and allowed to adhere for 24 h at 37 °C and 5% CO_2_. Subsequently, 0.1, 1, and 10 µM of free DOX, DOX-CTN4, and DOX-CTN4-HA were added, and cells were cultured for 4 h, washed, and further incubated for a total of 72 h. Untreated cells served as controls. At the end of the incubation time, 100 µL of MTT solution (5 mg/mL) was added to each well and incubated for an additional 4 h. After removal of the medium, a solubilization solution (16% SDS in 40% DMF, pH 4.7) was added to dissolve the formazan crystals. The plate was further incubated for 30 min at 37 °C. The absorbance of the formazan product was measured at 570 nm using an ELX800 microplate reader (BioTek Instruments, Inc., Winooski, VT, USA) in triplicate. The percentage of viable cells was determined using the following equation:

(2) $$Cell\;Viability=\frac{AT}{AU}\times 100$$

where AT is the absorbance of the treated cells and AU is the absorbance of the untreated cells. Cell viability values were expressed as the means of at least three different experiments ± SD.

### 3.8. Uptake Study

The CD44-positive human cancer cell line HeLa and the CD44-negative murine fibroblast cell line (NIH-3T3) were used to test the uptake of the cationic niosomes, uncoated and coated with HA. For this purpose, cells were incubated for 4 h with free DOX, DOX-CTN4, and DOX-CTN4-HA 1 μM each in culture medium. In particular, the cells were seeded on glass coverslip polylisine treated at 2 × 10^4^ cells/mL density and cultured in 6-well dishes with DMEM medium supplemented with 10% FBS, 2 mM l-glutamine, 100 IU/mL penicillin, and 100 µg/mL streptomycin. Afterward, the cells were rinsed three times with PBS to remove residual nanoparticles, fixed by paraformaldehyde 4% in PBS for 10 min, and rinsed again. Finally, the coverslip was mounted on a microscope slide with mounting medium containing n-propyl-gallate (5%) in glycerol and PBS, and then the cells were observed by a conventional fluorescence microscope (Microphot-FX, Nikon, Switzerland) both in phase contrast and fluorescence at 590 nm (488 ex). Images were taken, after about 5 min from microscope slide preparation, using a Canon EOS 1300D camera (Canon Inc., Tokyo, Japan) and ImageJ 1.53u software was used for fluorescence and phase contrast image merging.

## 4. Conclusions

By increasing the amount of cationic surfactant DODAB, this study obtains cationic niosomes. Dispersions of niosomes with a positive zeta potential can only be obtained with a ratio of R moles between the DDAB and the other components of the bilayer that is greater than 0.20. We focus exclusively on the preparation of CTN4, which has a PZ of 40 mV and a diameter of approximately 450 nm and can be stored for up to 3 months. The CTN4 formulation has also been coated with HA in order to obtain a target formulation, CTN4-HA, which has an average size of 453 nm and a PZ of −38 mV. Both formulations were used to deliver Doxorubicin and the developed niosomal systems showed a high drug loading capacity, with an encapsulation efficiency of approximately 80%. In addition, drug release from the niosomes was significantly slower than that from the DOX solution.

The results obtained indicate that, in the in vitro experimental conditions used, the HA-coated niosomal formulation (DOX-CTN4-HA) exhibited greater tumor-targeting ability and enhanced antiblastic activity than to the uncoated formulation. Moreover, the DOX-CTN4-HA formulation showed a lower hemolytic activity compared to DOX-CTN4. These findings suggest, in our opinion, that this type of nanoparticle is a highly promising candidate for targeted therapeutic applications.

## Figures and Tables

**Table 1:** Composition and physical-chemical characterization of empty niosomes in term of size (nm), PI, and ζ-potential.

Sample	SPAN80 (mg)	DODAB (mg)	CHOL (mg)	R Moles DODAB/(S80 + CHOL	Size (nm)	PI	ζ-potential (mV)
CTN0	10.0 ± 0.2	-	10.0 ± 0.2	0.00	508.1 ± 5.4	0.293	−39.7 ± 2.25
CTN1	10.0 ± 0.2	4.0 ± 0.3	12.0 ± 0.2	0.12	479.5 ± 2.3	0.262	−39.3 ± 0.98
CTN2	10.0 ± 0.2	6.0 ± 0.3	13.0 ± 0.2	0.17	575.7 ± 3.6	0.194	−13.2 ± 1.33
CTN3	10.0 ± 0.2	8.0 ± 0.2	14.0 ± 0.2	0.21	431.1 ± 3.8	0.224	+39.2 ± 0.78
CTN4	10.0 ± 0.2	8.0 ± 0.2	10.0 ± 0.2	0.26	452.1 ± 4.7	0.188	+40.0 ± 1.04

**Table 2:** Stability of CTN4 niosome stored at room temperature evaluated by measuring diameter, P.I., and ZP. Data were collected at specific time points, up to 4 months, and expressed as mean of three independent experiments ± SD.

Time (days)	Size (nm)	PI	ζ-potential (mV)
0	452 ± 11	0.192	+40.0 ± 1.04
30	478 ± 12	0.198	+41.0 ± 0.56
60	470 ± 18	0.239	+42.4 ± 1.42
90	488 ± 15	0.223	+42.1 ± 1.56
120	476 ± 10	0.154	+39.0 ± 1.33

**Table 3:** Physical characterization of cationic niosomes stored at 4 °C in terms of size (nm), PI, and ZP at 25 °C and expressed as mean of three independent experiments ± SD.

FORMULATION	Size (nm)	PI	ζ -Potential (mV)
CTN4	452 ± 4	0.192	+43.9 ± 0.7
CTN4-HA	546 ± 16	0.206	−37.7 ± 0.6
DOX-CTN4	473 ± 52	0.239	+45.5 ± 1.5
DOX-CTN4-HA	584 ± 51	0.232	−24.4 ± 0.4

## Data Availability

The data presented in this study are available upon request from the corresponding author.

## Supplementary Materials

The following supporting information can be downloaded at: www.mdpi.com/article/molecules30051148/s1, Figure S1: Size distribution and zeta potential of formulations: A- CTN4 and B-CTN4-HA; Figure S2: Curve calibration of Dox at different pH at 495 nm.
