# Supplementary material for: The Impact of Hyaluronic Acid Coating on the Cationic Niosomal Surface for Doxorubicin Delivery
Source: Molecules. 2025 Mar 3;30(5):1148. doi: 10.3390/molecules30051148 (PMC11901725; doi:10.3390/molecules30051148)

The Impact of Hyaluronic Acid Coating on the Cationic Niosomal Surface for Doxorubicin Delivery

Elisabetta Mazzotta [1], Martina Romeo [1], Giuseppina Sacco [1], Selene De Benedittis [2], Antonio Qualtieri [2], Ida Daniela Perrotta [3] and Rita Muzzalupo [1]

[1] Department of Pharmacy, Health and Nutritional Sciences, University of Calabria, 87036 Rende, Italy;
mazzotta-elisabetta@libero.it (E.M.); martina.romeo@unical.it (M.R.); giusysacco27@gmail.com (G.S.)

[2] Institute for the Research and the Biomedical Innovation (IRIB)-CNR-Mangone (CS), 00185 Rome, Italy; selene.db90@gmail.com (S.D.B.); antonio.qualtieri@cnr.it (A.Q.)

[3] Centre for Microscopy and Microanalysis (CM2), Department of Biology Ecology and Earth Sciences,
University of Calabria, 87036 Arcavacata di Rende, Italy; ida.perrotta@unical.it

* Correspondence: rita.muzzalupo@unical.it

Figure S1: Size distribution and Zeta Potential of formulations: A- CTN4 and B-CTN4-HA

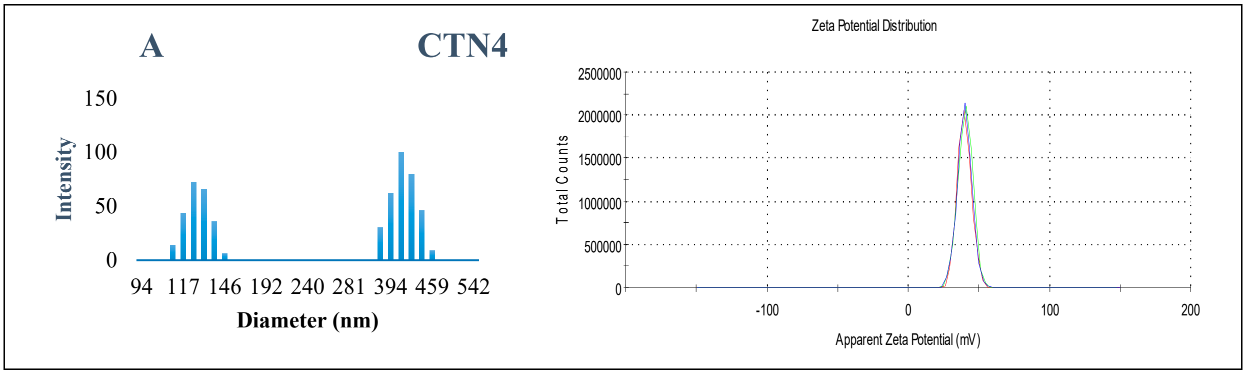

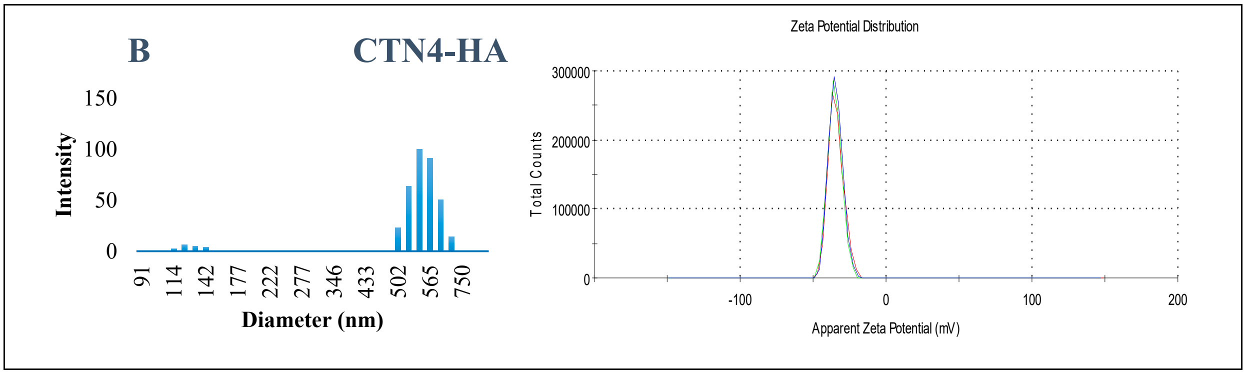

Figure S2: Curve calibration Dox at different pH at 495 nm

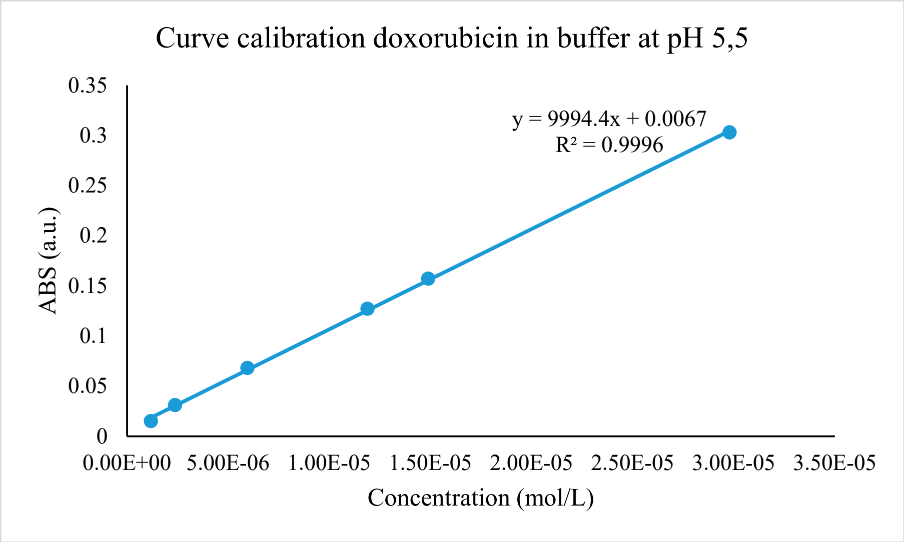

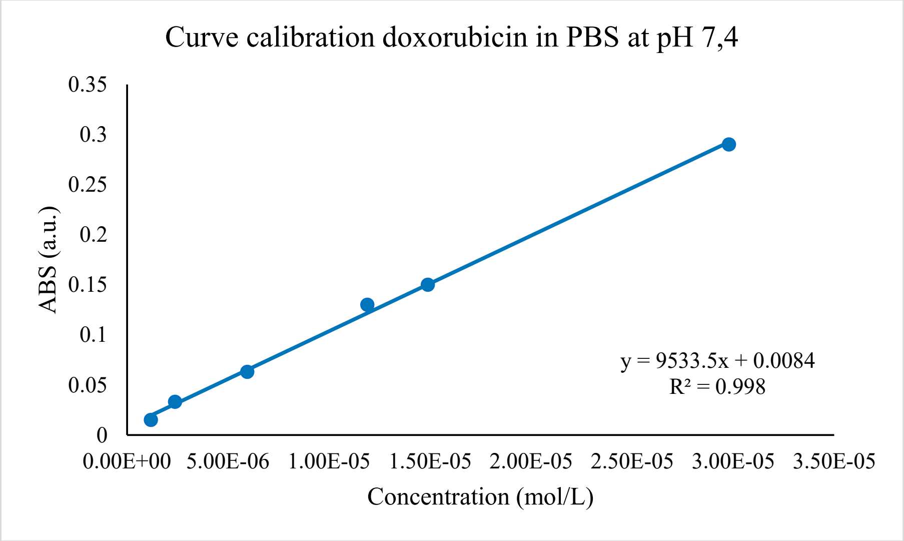

Supplement: Supplementary file 1 [file molecules-30-01148-s001.zip › molecules-3452089-supplementary.pdf]
